# The lncRNA *male-specific abdominal* plays a critical role in *Drosophila* accessory gland development and male fertility

**DOI:** 10.1371/journal.pgen.1007519

**Published:** 2018-07-16

**Authors:** Robert K. Maeda, Jessica L. Sitnik, Yohan Frei, Elodie Prince, Dragan Gligorov, Mariana F. Wolfner, François Karch

**Affiliations:** 1 Department of Genetics and Evolution, University of Geneva, Geneva, Switzerland; 2 Department of Molecular Biology and Genetics, Cornell University, Ithaca, New York, United States of America; The University of North Carolina at Chapel Hill, UNITED STATES

## Abstract

Although thousands of long non-coding RNAs (lncRNA) have been identified in the genomes of higher eukaryotes, the precise function of most of them is still unclear. Here, we show that a >65 kb, male-specific, lncRNA, called *male-specific abdominal* (*msa*) is required for the development of the secondary cells of the *Drosophila* male accessory gland (AG). *msa* is transcribed from within the *Drosophila* bithorax complex and shares much of its sequence with another lncRNA, the *iab-8* lncRNA, which is involved in the development of the central nervous system (CNS). Both lncRNAs perform much of their functions via a shared miRNA embedded within their sequences. Loss of *msa*, or of the miRNA it contains, causes defects in secondary cell morphology and reduces male fertility. Although both lncRNAs express the same miRNA, the phenotype in the secondary cells and the CNS seem to reflect misregulation of different targets in the two tissues.

## Author summary

In many animals, the male seminal fluid induces physiology changes in the mated female that increase a male’s reproductive success. These changes are often referred to as the post-mating response (PMR). In *Drosophila*, the seminal fluid proteins responsible for generating the PMR are made in a specialized gland, analogous to the mammalian seminal vesicle and prostate, called the accessory gland (AG). In this work, we show that a male-specific, long, non-coding RNA (lncRNA), called *msa*, plays a critical role in the development and function of this gland, primarily through a microRNA (miRNA) encoded within its sequence. This same miRNA had previously been shown to be expressed in the central nervous system (CNS) via an alternative promoter, where its ability to repress homeotic genes is required for both male and female fertility. Here, we present evidence that the targets of this miRNA in the AG are likely different from those found in the CNS. Thus, the same miRNA seems to have been selected to affect *Drosophila* fertility through two different mechanisms. Although many non-coding RNAs have now been identified, very few can be shown to have function. Our work highlights a lncRNA that has multiple biological functions, affecting cellular morphology and fertility.

## Introduction

Recent studies have shown that the genomes of many higher eukaryotes contain a large number of non-coding transcripts [1] [2] [3] [4] [5] [6]. Elucidating the function of these “non-coding” transcripts is now the topic of intense research. So far, much of the research done on these non-coding RNAs (ncRNAs) has concentrated on one class of small ncRNAs, called microRNAs (miRNAs). miRNAs are short, 22-nucleotide-long RNAs that guide Argonaute family proteins to target mRNAs via anti-sense base-pairing in order to repress the mRNAs’ expression post-transcriptionally [7–9]. Hundreds of miRNAs are encoded in the genomes of most, higher eukaryotes (~400 in humans, 140 in *Drosophila*, 110 in C. elegans), and many miRNAs are conserved through evolution [8]. Although it is estimated that >60% of protein-coding transcripts are regulated by at least one miRNA [10], removal of individual miRNAs rarely show an overtly visible phenotype [11, 12] [8, 13, 14]. Recent studies in *Drosophila*, however, has shown that the removal of many miRNAs result in subtle behavioral phenotypes that had previously gone unnoticed [15] [16]. It is now clear that, as a family, miRNAs can play subtle but important roles in a diverse number of processes ranging from development to aging [17–21].

**Fig 1:**
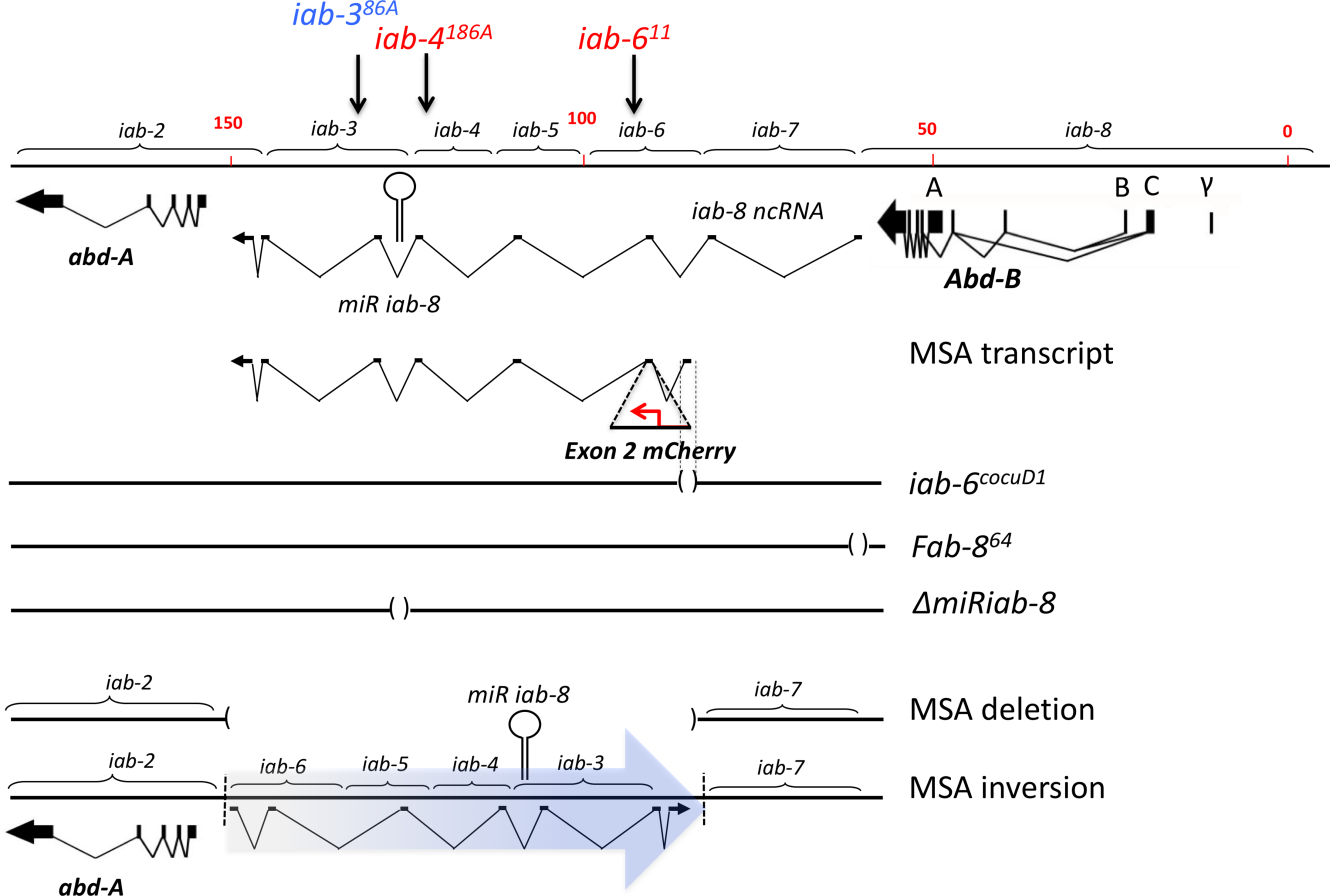
Mutations in the bithorax complex used in this study. The top line shows a scale map of the region of the *Drosophila bithorax complex* from *abd-A* to *Abd-B* with the parasegment specific cis-regulatory domains bracketed above the line. The exonic structure of the *abd-A* and *Abd-B* primary transcripts are shown as broken arrows below the line, connected with lines that indicate their splice patterns. Shown as a thinner broken arrow under the bithorax complex DNA is the most prominent splice version of the *iab-8 ncRNA*. The expected *msa* transcript is shown beneath the *iab-8* transcript. Under the primary transcripts, are some of the mutations used in this work. Deletions are indicated by () separating two horizontal lines. The *msa* inversion is also shown and labeled accordingly. The arrows above the bithorax map indicate the positions of chromosomal rearrangement breaks used in this work. The chromosomal breaks are label and are color-coded (red for breaks that do not complement *Df(P9)* in the accessory gland, and blue for breaks that complement the *Df(P9)* in the accessory gland).

Another class of ncRNAs is comprised of non-coding transcripts with lengths of more than 200 nucleotides, called long non-coding RNAs (lncRNAs) [2, 22, 23]. Even though lncRNAs represent the largest class of non-coding transcripts [1], with a few exceptions, the functions of most lncRNAs remain largely unknown [22, 23]. Genetic manipulation of model organisms has proven to be a powerful tool to discover the functions of ncRNAs [12]. Previously, we, and others, characterized a particular lncRNA in *Drosophila*, the *iab-8 lncRNA* [24–29]. This lncRNA is transcribed from the *Drosophila* bithorax complex, primarily in the posterior central nervous system, beginning in early development. It is over 90kb in length and is both spliced and polyadenylated (Fig 1). One function of this lncRNA is to act as a template for the production of a miRNA that is encoded within its intronic sequence. This miRNA, *miR-iab-8*, has been characterized as primarily targeting the homeotic genes, *abd-A* and *Ubx*, along with their cofactors, *hth* and *exd* [24, 26, 27, 29]. The biological consequence of the loss of the *iab-8* lncRNA is male and female sterility, thought to be due to defects in the innervation of the abdominal and/or reproductive tract muscles of the fly [24]. These reproductive defects were shown to be the result of overexpression of the Hox targets of the *iab-8* miRNA [9, 29].

In 2011, Graveley et al, described a transcript that appeared to be a variant of the *iab-8* lncRNA. This lncRNA starts from a promoter located just downstream of the *Fab-7* boundary (within the *iab-6 cis*-regulatory domains that controls *Abd-B*) and, like the *iab-8* lncRNA, also contains the precursor of *mir-iab-8* [30]. As this transcript was expressed exclusively in the adult male abdomen, they named this lncRNA *male-specific abdominal* (*msa*) (Fig 1). Here, we show that *msa* is expressed in the secondary cells of the male accessory gland and that its function is required for secondary cell development and maximal male fecundity.

Like the mammalian seminal vesicle and prostate gland, the *Drosophila* male accessory glands make many important components of the seminal fluid [31]. Each gland is composed of a monolayer of secretory cells surrounding a central lumen. There are two types of binucleate secretory cells in the accessory gland. Ninety-six percent of the cells are “main cells”; the remaining four percent are “secondary cells” [32] [33].

Seminal fluid proteins that are produced by the accessory gland increase male reproductive success by inducing post-mating responses (PMR) in mated females. The PMR is the suite of the behavioral and physiological changes that occur in the female after mating, and include, among many other things, an increase in egg laying/production and a rejection of the courtship by subsequent males [31, 34]. PMR phenotypes that last longer than the first ~2 days post-mating are called the long-term PMR (LTR). Recently, we, and others, showed that the secondary cells produce proteins that are essential for the LTR [35, 36] [37, 38].

Here, we report that the *msa* lncRNA is expressed in the secondary cells. We show that expression of this lncRNA, and of the miRNA (*miR-iab-8*) encoded in one of its introns, is required for secondary cell development and thus for the male’s ability to induce long-term post-mating responses in his mate. Interestingly, the major targets for this miRNA in the secondary cells do not include some of the miRNA’s known targets in the CNS. Thus, a single miRNA plays two roles in the process of male fertility but probably through two different mechanisms in the two tissues.

## Results

### The *msa* variant of the *iab-8* lncRNA is expressed in the secondary cells

Graveley et al. (2011) isolated a male-specific transcript from abdominal tissue whose promoter mapped to the *iab-6* domain of the *Drosophila* bithorax complex [30]. Examining this sequence more carefully, we discovered that the first exon of *msa* fell within an enhancer region that we previously demonstrated to be required for *Abd-B* expression in the secondary cells of the male accessory gland (Fig 1) [35]. Loss of this enhancer (*iab-6*^*cocu*^) eliminates ABD-B expression from these cells and causes both cytological and reproductive phenotypes [35, 36]. We were able to confirm the role played by *Abd-B* in this process by showing that the *iab-6*^*cocu*^ phenotype could be partially rescued using an *Abd-B* expressing transgene and that an *Abd-B* RNAi construct was able to create an *iab-*6^*cocu*^-like phenotype when expressed in the secondary cells. Interestingly, in those experiments both the rescue and RNAi-induced phenotype were weaker than expected [35]. As the *iab-6*^*cocu*^ mutation deletes the promoter and first exon of *msa*, we asked if the incomplete nature of both the rescue and RNAi phenotype could be due to a role of the *msa* lncRNA (see Fig 1).

**Fig 2:**
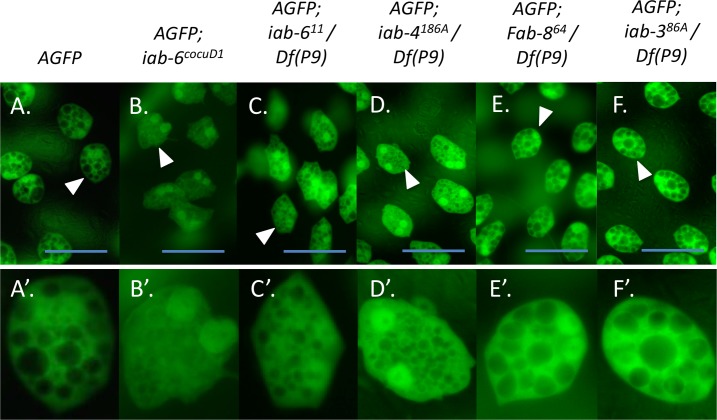
A lncRNA is plays a role in the development of the accessory gland. Accessory gland phenotypes associated with BX-C mutations. In each case, a chromosome (here called *AGFP)* containing a BAC transgene containing the *Abd-B* regulatory sequence driving Gal-4 expression specifically in the secondary cells and a UAS-nGFP transgene was used to visualize the secondary cell cytoplasm and nuclei (more concentrated GFP). Vacuoles can be seen as black spaces within the cell. Genotypes are labeled above each panel. ***A*.** A wild-type accessory gland. ***B*.** *iab-6*^*cocuD1*^*/ iab-6*^*cocuD1*^. The rest of the panels are hemizygous for the BX-C mutants described in Fig 1: ***C*.** *iab-6*^*11*^*/Df(3R)P9*, ***D*.** *iab-4*^*186A*^*/Df(3R)P9*, ***E*.** *Fab-8*^*64*^*/Df(3R)P9 and* ***F*.** *iab-3*^*86A*^*/Df(3R)P9*. Note the smaller vacuoles, similar to ***B*.** (*iab-6*^*cocuD1*^*/ iab-6*^*cocuD1)*^ in ***C*.** (*iab-6*^*11*^*/Df(3R)P9) and* ***D*.** *(iab-4*^*186A*^*/Df(3R)P9)*. Scale bar equals 50μm. ***A’*.*-F’*.** are enlargements of individual secondary cells from the ***A*.*-F*.** White arrow heads mark the cells that were enlarged.

The *iab-6*^*cocuD1*^ mutation is a 1.1kb deletion of the *iab-6*^*cocu*^ enhancer (Fig 1) and is the smallest *iab-6*^*cocu*^ deletion that we have made. Secondary cells of *iab-6*^*cocuD1*^ males display abnormal cytological phenotypes (Figs 2B and S2B) and lack ABD-B expression (see S1 Fig). In wild type accessory glands, the secondary cells have a characteristic morphology: they are round and contain a substantial number of large vacuolar structures within their cytoplasm (Figs 2A and S2A) [33] [35]. In *iab-6*^*cocuD1*^ males, the secondary cells are less round (often appearing more hexagonal (main cell-like) in shape) and lose or severely reduce the size of the vacuolar structures (Figs 2B and S2B). In order to functionally test if the *msa* transcript is made in the secondary cells and to determine if its loss results in a secondary cell phenotype, we tested if mutations that should disrupt the *msa* transcription unit led to morphological phenotypes in the secondary cells. To do this, we examined the accessory glands of flies containing different BX-C mutations (*Fab-8*^*64*^, *iab-6*^*11*^, *and iab-4*^*186*^) [39] [40] [41] over a deficiency of the whole BX-C [*Df(3R)P9*] [42] (Figs 1, 2 and S2). Two mutations (*iab-4*^*186A*^ *and iab-6*^*11*^) are chromosomal rearrangements with breaks that lead to truncated versions of the *msa* transcript. The *Fab-8*^*64*^ mutation removes the previously characterized promoter of the *iab-8* lncRNA and should indicate if the larger *iab-8* lncRNA is functionally important in the secondary cells [27]. To more easily visualize the potential phenotypes, all experiments were performed in the presence of a GFP reporter that is secondary cells specific in the AG (*Abd-B-Gal4*, *UAS-GFP* referred as to *AGFP* in Fig 2). In secondary cells, this reporter fills the nuclei and cytoplasm with GFP, but is excluded from the vacuoles [35].

Our results show that both the *iab-6*^*11*^*and iab-4*^*186*^ breaks show variably expressed defects in secondary cell morphology (vacuolar structures become smaller) when placed over a BX-C deficiency (Figs 2C, 2D, S2C and S2D) but that secondary cells from *Fab-8*^*64*^/ *Df(3R)P9* males appear normal (Figs 2E and S2D). The fact that the *Fab-8*^*64*^ mutation did not have an effect on secondary cell morphology (Fig 2E) indicates that the full-length *iab-8* lncRNA probably does not play a critical role in secondary cell development. However, the effects of the *iab-6*^*11*^*and iab-4*^*186*^ breaks are consistent with the *msa* lncRNA playing a crucial role in secondary cell development.

**Fig 3:**
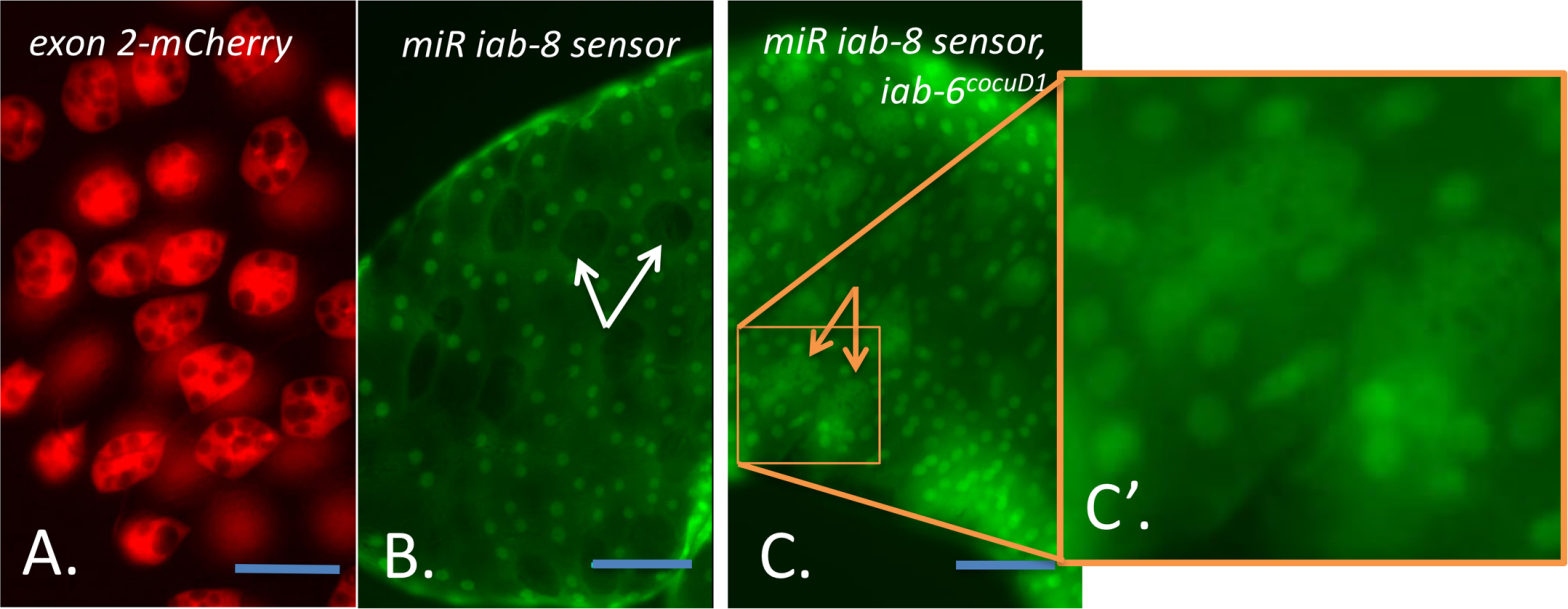
Detection of miR iab-8 using a GFP sensor. Dissected accessory glands from ***A*.** a fly carrying an mCherry reporter in the *msa* second exon (*exon2-mCherry*), ***B*.** a wild type fly carrying a *mir iab-8* GFP sensor and ***C*.** an *iab-6*^*cocuD1*^ homozygous fly in the presence of a *mir iab-8* GFP sensor (22)**. *C’*.** is an enlargement of the box shown in ***C*.** In ***A*.**, mCherry is visualized directly, while in B., C. and C’. GFP is visualized. ***B*.** In wild-type AGs, secondary cells appear as non-GFP expressing holes in the plane of GFP-expressing main cells (examples shown with white arrows). ***C*.** In the *iab-6*^*cocuD1*^ homozygous mutants, the secondary cells express GFP (examples shown with orange arrows). B’ is an enlargement of the orange box in B. The enlargement of the secondary cells allows for the visualization of the abnormal morphological phenotype (lack of large vacuoles) of *iab-6*^*cocuD1*^ secondary cells in the sensor line. Scale bar equals 50μm.

To verify that the *msa* transcript is indeed expressed in the secondary cells, we placed an mCherry reporter within its second exon, and examined mCherry expression. Throughout the lifecycle of transgenic flies carrying this reporter, we detected mCherry only within the secondary cells of the adult male’s AG (Fig 3A), indicating that *msa*’s expression is secondary cell-specific.

As the *msa* transcript shares most of its sequence with the *iab-8* lncRNA, it should contain the precursor of the *miR-iab-8* miRNA between its fifth and sixth exons. We reasoned that if the *msa* lncRNA is important for secondary cell morphology then some of that function could be due to expression of *miR-iab-8*. If this were so, then we would expect that mutations that truncate the *msa* transcript downstream of the miRNA should show little or no morphological defects. To test this, we examined the *iab-3*^*86A*^ break, which breaks the transcript just downstream of the miRNA (Fig 1). Flies carrying the *iab-3*^*86A*^ break over a BX-C deficiency do not show an *iab-6*^*cocuD1*^–like secondary cell morphological phenotype (small or missing vacuoles), suggesting that *miR-iab-8* or an element located between the *iab-3*^*86A*^ and the *iab-4*^*186*^ breakpoints, is critical for secondary cell morphology (Figs 2F and S2F).

To show that *miR-iab-8* is expressed in secondary cells, we used a GFP miRNA sensor, specifically designed to detect *miR-iab-8* [25] [29]. This miRNA sensor contains binding sites for *miR-iab-8* in the 3’UTR of a ubiquitously expressed GFP reporter. Thus, in cells in which *miR-iab-8* is expressed, the GFP reporter is silenced, leading to GFP-negative cells in an otherwise GFP-positive background. Examining the secondary cells of flies carrying the GFP sensor showed that within the AG, only the secondary cells lack GFP expression. This indicates that *miR-iab-8* is indeed expressed in the secondary cells of the male accessory gland (Fig 3B). We next tested if the GFP sensor is affected in the *iab-6*^*cocuD1*^ mutation that removes the *msa* promoter and first exon. As seen in Fig 3C, *iab-6*^*cocuD1*^ mutants display high levels of GFP expression throughout the accessory gland, indicating that *miR-iab-8* expression is abrogated or severely impaired in these mutant cells.

From these results, we conclude that the *msa* lncRNA is expressed in the secondary cells of the male AG through its promoter in the *iab-6*^*cocuD1*^ region. Furthermore, given that we previously showed that deletions removing a large portion of the *msa* transcript upstream of the miRNA (but leaving its promoter intact, like *iab-4*,*5*,*6*^*DB*^ [35] [43]) causes no noticeable secondary cell phenotype, we believe that *msa* probably fulfills most of its function in these cells via the *miR-iab-8* miRNA embedded within its sequence.

### *Abd-B* and *miR-iab-8* play complementary roles in secondary cell development

**Fig 4:**
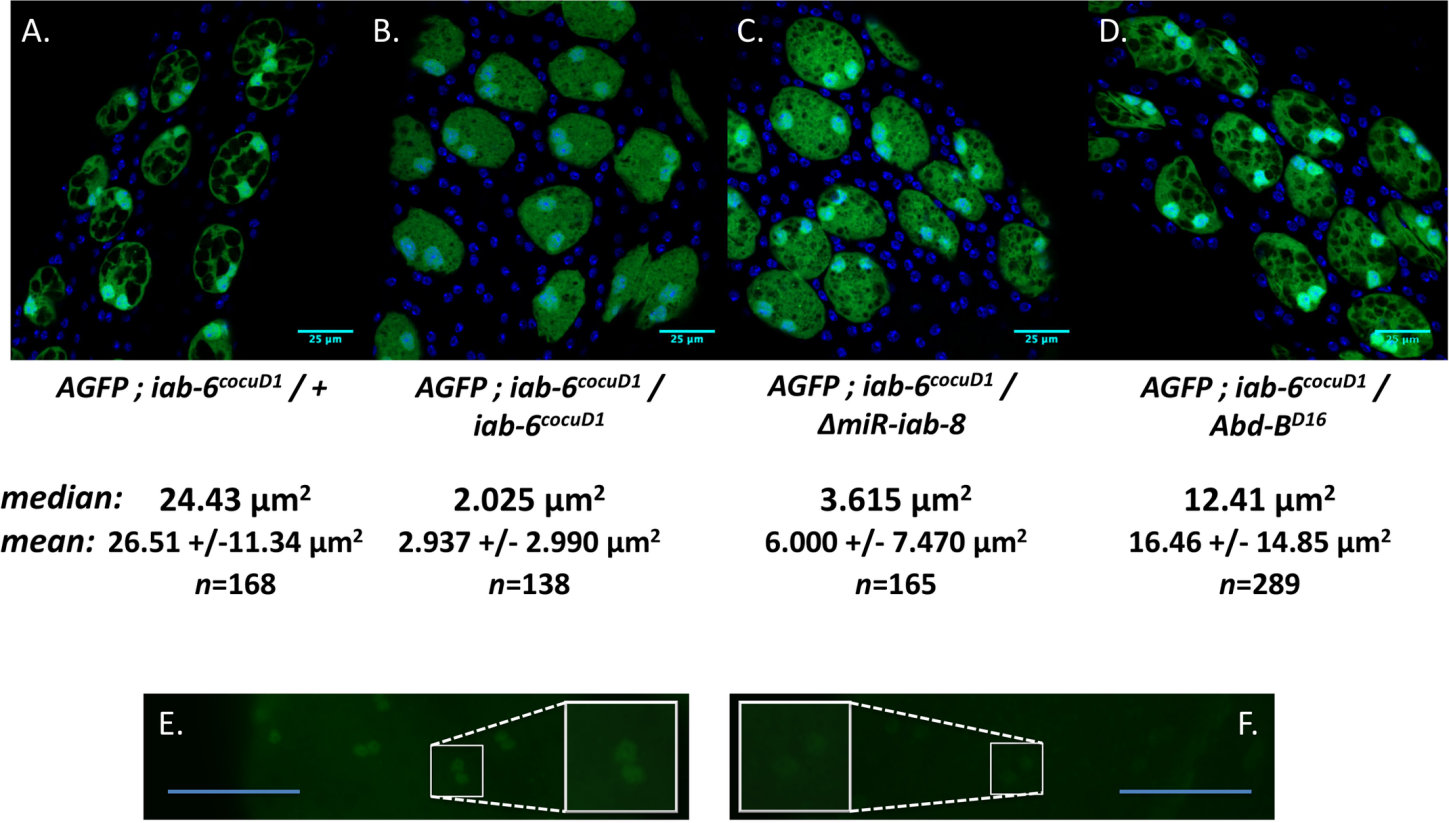
Loss of the iab-8 miRNA and/or Abd-B leads to defects in vacuole formation. Accessory glands are shown from three to four-day old virgin males that express the *Abd-Gal4*, *UAS-GFP* reporter and carry the following mutations: ***A*.** *iab-6*^*cocuD1*^*/+* (control), ***B*.** *iab-6*^*cocuD1*^*/ iab-6*^*cocuD1*^(*MSA*^*-*^, *Abd-B*^*-*^), ***C*.** *iab-6*^*cocuD1*^*/ΔmiR-iab-8* (*miRNAiab-8*^*-*^) *and* **D.** *iab-6*^*cocuD1*^*/Abd-B*^*D16*^ (*Abd-B*^*-*^). Below panels ***A*.*-D*.** are the median and mean vacuole sizes for each genotype along with the standard deviation for the mean vacuole size and the number of vacuoles measured (*n*). Based on Kruskal-Wallis one-way analysis of variance, followed by *post-hoc* Dunn’s tests, the differences in values is significant between all groups (p < .0001 for all pairwise comparisons except *iab-6*^*cocuD1*^ homozygotes *vs iab-6*^*cocuD1*^*/miR-iab-8* mutants, where p = .0085. Figures ***E*.** and ***F*.** show AGs stained with antibodies to ABD-B (green), imaged using the same settings. In ***F*.,** the AG is from *iab-6*^*cocuD1*^*/Abd-B*^*D16*^ males (where *Abd-B* should not be expressed), and in ***E*.** the AG is from a sibling control of the cross, *iab-6*^*cocuD1*^*/Dp(P5)* (where two wild-type copies of *Abd-B* should be expressed). An enlargement of a single secondary cell is present in each panel to show the difference in *Abd-B* expression level in the two lines. Scale bars = 25μm for ***A*.*-D*.,** and = 50μm for ***E*.** and ***F***.

Our findings suggest that the *iab-6*^*cocuD1*^ deletion is actually a double mutant in the sense that it affects both *Abd-B* and *msa/miR-iab-8* expression. We thus wanted to know how much of the *iab-6*^*cocuD1*^ phenotype is due to the loss of *miR-iab-8*. Because homozygous *miR-iab-8* mutations cause sterility due to a role in the CNS and affects gene expression in multiple tissues [24], we examined the effects of the loss of *miR-iab-8* specifically in secondary cells by placing a *mir-iab-8* deletion in *trans* to *iab-6*^*cocuD1*^. Previous data from our lab indicates that the *miR-iab-8* deletion should leave the *msa* transcript intact; the miRNA precursor is located inside an intron and the closely related *iab-8* lncRNA is still made in *miR-iab-8* deletion lines [27]. Therefore, these transheterozygotes should be mutant for *miR-iab-8* in the secondary cells but still express ABD-B (and the rest of the *msa* transcript) (S1E Fig). To make any resulting phenotypes easier to see, we again included a secondary cell-specific GFP marker in all flies. The results of these experiments can be seen in Fig 4.

Our results indicate that loss of *miR-iab-8* alone significantly impairs secondary cell development, but not as severely as the *iab-6*^*cocuD1*^ mutation itself [Fig 4B and 4C; median vacuole size (area of the vacuole at its widest point) control = 24.43 μm^2^, *iab-6*^*cocuD1*^ homozygotes = 2.025 μm^2^, *iab-6*^*cocuD1*^*/miR-iab-8* = 3.615 μm^2^. The two mutant genotypes are significantly different from the control (p < .0001 (Kruskal-Wallis one-way analysis of variation followed by a *post hoc* Dunn’t test) [44, 45]) and from each other (p = .0085)]. We also assessed the contribution of *Abd-B* by crossing an *Abd-B* null allele (*Abd-B*^*D16*^) to *iab-6*^*cocuD1*^. These males also showed reduced vacuole size (Fig 4D) (12.41 μm^2^ relative to heterozygous cells (p < .0001)), though the effects were milder than those of either *iab-6*^*cocuD1*^ homozygotes or *iab-6*^*cocuD1*^*/miR-iab-8* mutants (p < .0001). These results indicate that *Abd-B* may play a lesser role in secondary cell development. However, the *Abd-B* locus is a region of the genome that is highly susceptible to transvection events [46] [47] [48]. Indeed, it seems that within the secondary cells, some transvection is occurring, as we observe low but detectable ABD-B levels in *iab-6*^*cocuD1*^*/ Abd-B*^*D16*^ secondary cells (Fig 4E and 4F). Thus, the *iab-6*^*cocuD1*^*/Abd-B*^*D16*^ phenotype may be mitigated by interactions between the *Abd-B* enhancers on the *Abd-B*^*D16*^ chromosome with the *Abd-B* promoter on the *iab-6*^*cocuD1*^chromosome.

**Fig 5:**
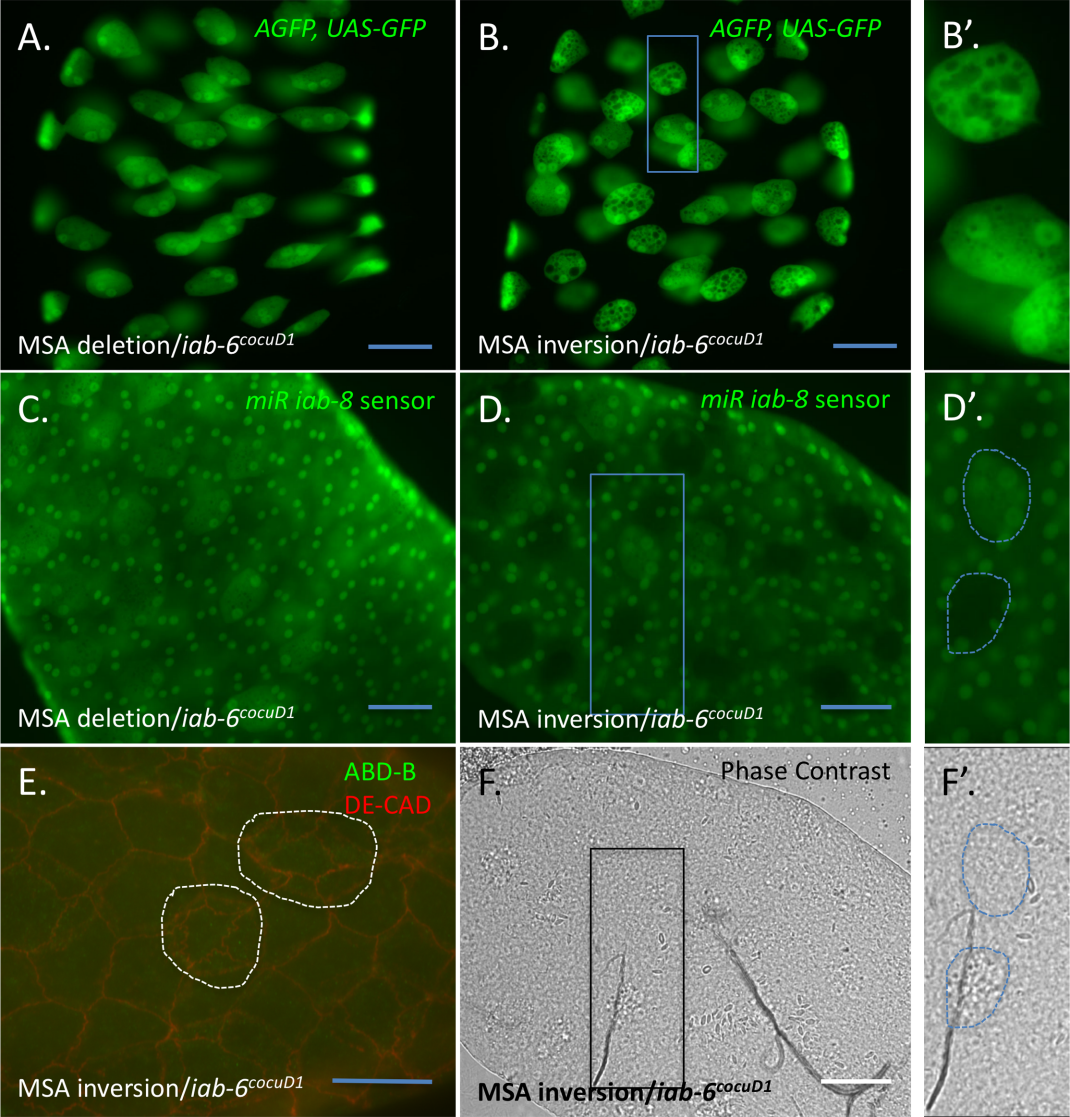
CrispR-mediated deletions and inversions of msa confirm the role of ABD-B in the accessory glands. ***A*.** Accessory glands from *iab-3*,*6*^*CR6YO1M20M2*^ (*msa* deletion) / *iab-6*^*cocuD1*^ in the presence of the *Abd-B-Gal4*, *UAS-GFP* reporter show a phenotype like that of *iab-6*^*cocuD1*^ homozygotes. ***B*.** Accessory glands from *iab-3*,*6*^*CR6YO1M20M1*^ (*msa* inversion) / *iab-6*^*cocuD1*^ in the presence of the *Abd-B-Gal4*, *UAS-GFP* reporter show a mixed phenotype with some cells resembling *iab-6*^*cocuD1*^ homozygotes and other cells being less affected. This can be seen more easily in ***B’*.**, which is an enlargement of the rectangle indicated in ***B*. *C*.** Accessory glands from *iab-3*,*6*^*CR6YO1M20M2*^ (*msa* deletion) / *iab-6*^*cocuD1*^ in the presence of the *iab-8* miRNA sensor shows that the *iab-8* miRNA is not present. ***D*.** Accessory glands from *iab-3*,*6*^*CR6YO1M20M1*^ (*msa* inversion) / *iab-6*^*cocuD1*^ in the presence of the *iab-8* miRNA sensor show some cells expressing the miRNA while others do not. ***D’***. shows an enlargement of the rectangle present in ***D*.** with two secondary cells traced with a dashed blue line). ***F*.** and ***F’*.** are phase contrast images the same glands shown in ***D*.** and ***D’*.** From ***D’*.** and ***F’*.** one can see how cells expressing the miRNA retain vacuoles visible by phase contrast (as a rougher appearance), while the cells not expressing the miRNA do not (resembling *iab-6*^*cocuD1*^ homozygotes). ***E*.** ABD-B (green) and DE-Cadherin (red) immunostaining of glands from *iab-3*,*6*^*CR6YO1M20M1*^ (*msa* inversion) / *iab-6*^*cocuD1*^. ABD-B is absent in this mutant line. Scale = 50μm.

To further differentiate the contribution of the Abd-B relative to the miRNA in the *iab-6*^*cocuD1*^ phenotype, we examined two additional mutations of the msa transcript created using the CrispR-Cas9 system (Fig 1). Consistent with expectations, in a line where the *msa* transcript was completely deleted, both *Abd-B* and *miR-iab-8* (based on the miRNA sensor[25]) were completely undetectable in the accessory glands. The phenotype of males heterozygous for this allele validated our hypothesis for the function of the lncRNA: males carrying the *msa* deletion over *iab-*6^*cocuD1*^ showed a secondary cell phenotype similar to that of *iab-6*^*cocuD1*^ homozygotes, with no large vacuoles (Fig 5A).

In the course of generating the *msa* deletion, we fortuitously recovered a second mutation that let us further explore the role of the miRNA. This mutation is a near perfect inversion of the intervening 68kb sequence (Fig 1). Surprisingly, males carrying this inversion over *iab-6*^*cocuD1*^ displayed a variable secondary cell phenotype, with some cells looking like *iab-6*^*cocuD1*^ homozygotes and others showing a markedly weaker phenotype (some vacuoles, though mostly smaller) (Fig 5B and 5B’). *Abd-B* protein was undetectable in all secondary cells from these males (Fig 5E). Interestingly, the GFP-based *miR-iab-8* sensor [25] showed that some secondary cells expressed the miRNA, while others did not (Fig 5C and 5D). We then tested whether absence or presence of the miRNA correlated with the severe vs. weak phenotype. Because we could not use our normal GFP reporter to judge the relative severity of the phenotypes in the cells expressing the sensor, we used phase contrast microscopy. As seen in Fig 5F and 5F’, secondary cells that express the *iab-8* miRNA still have visible vacuoles (appearing as rougher patches within the cells under phase contrast), whereas no vacuoles are seen in secondary cells that do not contain the miRNA. These results lead to the conclusion that absence of the miRNA, in the absence of *Abd-B*, leads to a secondary cell phenotype like that seen in *iab-6*^*cocu*^ homozygotes, whereas the presence of the miR, in the absence of *Abd-B* leads to a milder phenotype (some small vacuoles). Overall, these results support our results using the *Abd-B*^*D16*^ mutation that suggested that Abd-B plays a significant, though perhaps lesser role in secondary cell development.

### The *iab-8* miRNA is required for the male’s capacity to generate a long-term post-mating response in his mate

After mating to wild type males, females exhibit characteristic changes in behavior called the post-mating response (PMR) (reviewed in [31]). These changes in behavior include an increase in egg production and deposition, as well as a decrease in receptivity to remating. Persistence of these changes for >1 day post-mating, is due, in part, to the action of secondary cell produced proteins [35]. To determine if the PMR changes seen in *iab-6*^*cocuD1*^ mutants are mediated by the *iab-8* miRNA, we compared the long-term post-mating responses (LTR) induced by *iab-6*^*cocu*^*/miR-iab-8* males to both *iab-6*^*cocu*^ homozygous males and heterozygous control males. We assessed the LTR using two assays: (1) we tested for the long-term increase in egg-laying and (2) we tested for the long-term decrease in remating receptivity. Because the quantitative levels of these phenomena can be sensitive to genetic background, we carried out assays on both *iab-6*^*cocuD1*^ and a second *iab-6*^*cocu*^ mutation, *iab-6*^*cocuD5*^ (the original *iab-6*^*cocu*^ deletion that is 2kb larger than *iab-6*^*cocuD1*^) [35].

**Fig 6:**
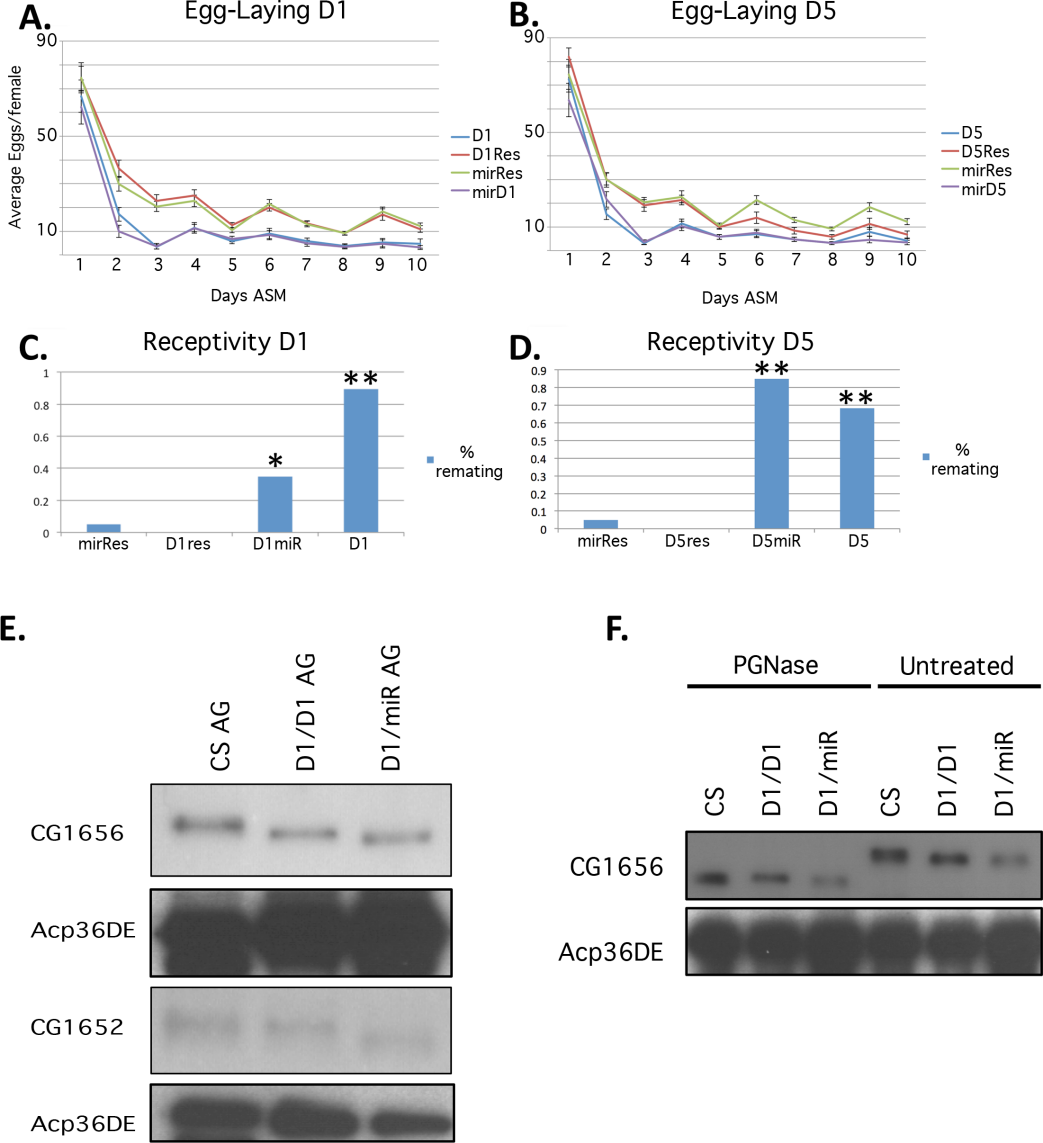
miR iab-8 loss-of-function causes defects in the LTR. Two different fertility fecundity assays were used to assess the LTR of mutants lacking the *iab-8* miRNA: (**A. and B.**) egg-laying over the 10 days post-mating and (**C. and D.**) receptivity after four days post-mating. The *ΔmiR-iab-8* mutation was tested over two different *iab-6*^*cocu*^ alleles to overcome possible effects of genetic background *iab-6*^*cocuD1*^(D1) *or iab-6*^*cocuD5*^ (D5). For the 10-day egg laying assay, in **A.**, the D1 curve (in blue, n = 15) refers to *iab-6*^*cocuD1*^homozygous males, the D1Resc curve (in red, n = 18) refers to *iab-6*^*cocuD1*^*/ iab-5*,*6*^*rescue*^ males, where *iab-5*,*6*^*rescue*^ is a chromosome made with the same InSiRT platform [70] used to make the *iab-6*^*cocu*^ mutations but with wild-type sequence added in its place. The mirResc curve (in green, n = 22) refers to *ΔmiR-iab-8/ iab-5*,*6*^*rescue*^ males and the mirD1 curve (in purple, n = 16) refers to *iab-6*^*cocuD1*^*/ ΔmiR-iab-8* males. In **B.**, the D5 curve (in blue, n = 18) refers to *iab-6*^*cocuD5*^ homozygous males, the D5Resc curve (in red, n = 22) refers to *iab-6*^*cocuD5*^*/ iab-5*,*6*^*rescue*^ males, the mirResc curve (in green, same as in **A.,** n = 22) refers to *ΔmiR-iab-8/ iab-5*,*6*^*rescue*^ males and the mirD5 curve (in purple, n = 21) refers to *iab-6*^*cocuD5*^*/ ΔmiR-iab-8* males. In both cases there is a significant drop in egg laying in mates of either *iab-6*^*cocu*^ mutant or in mates of males transheterozygous for either *iab-6*^*cocu*^ mutant and the *ΔmiR-iab-8* chromosome (rmANOVA p>0.001 relative to controls). The receptivity assay was performed on the same genotypes. In **C.** and **D.**, sample sizes are as follows, D1 (n = 19), D1 Resc (n = 18), mir Resc (n = 20) mirD1 (n = 20), D5 (n = 19), D5 Resc (n = 20), mir Resc (same as in **C.,** n = 20) and mirD5 (n = 20). In both cases, there is a significant increase in remating (p < .05) by mates of either *iab-6*^*cocu*^ mutants or in mates of males transheterozygous for either *iab-6*^*cocu*^ mutant and the *ΔmiR-iab-8* chromosome (* indicates p < .007 vs D1 res, p≤.02 mir Res and p < .001 D1 relative to controls, ** indicates p < .001 relative to controls, as accessed by the Wilcoxin Ranked Sums Test). ***E*.** Shows extracts from single AGs run on Western blots and probed with antibodies against CG1656 or CG1652. Below these blots are images of the same blots stripped and reprobed with a loading control antibody against the main cell protein, Acp36DE. The genotype of the flies from which the AGs were dissected are indicated above each lane and the antibodies used indicated on the left. ***F***. Shows western blots of extracts from wild type or mutant AGs probed with antibodies against CG1656. The extracts on the left were treated with PGNase F, while those on the right were not. Genotypes of the flies used for the extracts are indicated above each lane. Below the blot is an image of the same blot stripped and reprobed with a loading control antibody against the main cell protein, Acp36DE.

In the fertility/fecundity assay (Fig 6A and 6B), mates of transheterozygous *iab-6*^*cocu*^*/miR-iab-8* mutants showed a significantly decreased LTR relative to mates of heterozygous control males: mates of *miR-iab-8* deficient males laid significantly fewer eggs after the initial short-term increase in egg-laying. This phenotype in the mates of *iab-6*^*cocu*^*/miR-iab-8* transheterozygous males was identical to that of females mated to homozygotes for either of the *iab-6*^*cocu*^ mutants.

Results with the receptivity assay were also consistent with the finding that the LTR is disrupted in mates of *mir-iab8* transheterozygous males (Fig 6C and 6D.) Mates of either transheterozygous *iab-6*^*cocu*^*/miR-iab-8* mutants showed a significant increase in receptivity over heterozygous control males in the four-day receptivity assay, although there were slight numerical differences between the results with the two *iab-6*^*cocu*^ alleles; mates of *iab-6*^*cocuD5*^*/miR-iab-8* males showed a stronger effect than mates of *iab-6*^*cocuD1*^*/miR-iab-8*.

Our results indicate that *miR-iab-8* plays a major role in the male’s ability to induce the LTR in his mate. Previous RNAi results [35] showed that *Abd-B* also plays an important role in the male’s ability to induce an LTR. Thus, we conclude that the *iab-8* miRNA and *Abd-B* both play a role in the male’s ability to induce the LTR in his mate.

The PMR experiments highlight the role of the secondary cells in propagating the long-term response. A number of proteins have been implicated in regulating the LTR. Two of these proteins are CG1652 and CG1656 (annotated in flybase as Lectin-46Cb and Lectin-46Ca, repectively) [49, 50]. Previously, we showed that these proteins migrate differently on SDS-PAGE gels when isolated from *iab-6*^*cocu*^ mutants or wild-type AGs [35]. In order to test if this is also true for *iab-6*^*cocu*^*/miR-iab-8* transheterozygous males, we performed Western blot analysis on AG extracts from wild type, *iab-6*^*cocuD1*^ homozygous and *iab-6*^*cocuD1*^*/miR-iab-8* transheterozygous males. As seen in Fig 6E, the CG1652 and CG1656 proteins migrate faster when isolated from either *iab-6*^*cocuD1*^ homozygous males or *iab-6*^*cocuD1*^*/miR-iab-8* transheterozygous males relative to these proteins from extracts of wild type males. In fact, we noticed that both the CG1652 and CG1656 proteins seem to migrate slightly faster in extracts from *iab-6*^*cocu*^*/miR-iab-8* AGs than from *iab-6*^*cocuD1*^AGs (Fig 6E). In Gligorov et al., we showed that the genotype specific difference in CG1656 migration was due to changes in N-linked glycosylation; treatment of extracts with PGNase F led to proteins that migrated at equal velocities [35]. We repeated these treatments on extracts from *iab-6*^*cocuD1*^*/miR-iab-8* transheterozygous AGs. Although PGNase treatment equalized CG1656 migration from in *iab-6*^*cocuD1*^ and wild-type extracts, CG1656 from *iab-6*^*cocuD1*^*/miR-iab-8* transheterozygous AGs continued to migrate more rapidly (Fig 6F). These results suggest that there is a difference in secondary cell characteristics between the two mutant genotypes.

### CNS targets of the *iab-8* miRNA like *abd-A* and *Ubx*, are probably not the main targets of the *msa* lncRNA in the secondary cells

**Fig 7:**
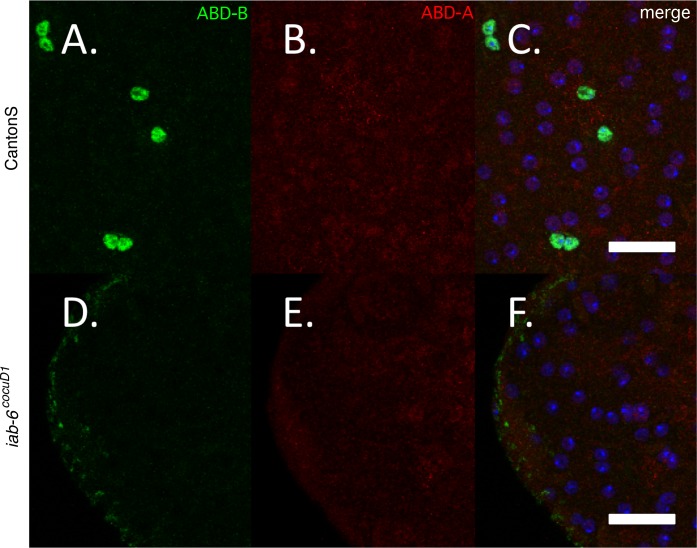
Known miR-iab-8 target abd-A is not overexpressed in iab-6^cocuD1^secondary cells. ABD-A staining in *wild-type* or mutant secondary cells. Accessory glands from **A.-C.** CantonS (*wild-type*), or **D.-F.** *iab-6*^*cocuD1*^*/ iab-6*^*cocuD1*^ males immunostained for ABD-B (in green **A. and D.**), ABD-A (in red, **B. and E.)** and DAPI (in the merged image in blue **C.** and **F.**). Scale = 50μm.

The strongest known target of the *iab-8* miRNA is the *abd-A* gene [24, 26, 27]. Indeed, in the embryonic CNS, it has been shown that the *iab-8 lncRNA* represses the expression of the *abd-A* gene through two mechanisms: through the *iab-8* miRNA and through apparent transcriptional interference at the *abd-A* promoter [27]. *iab-8 lncRNA* mediated *abd-A* repression in the CNS has been implicated in the female sterility phenotype associated with removal of the *iab-8 lncRNA* [27]. In order to determine if the *msa* transcript is important for regulating *abd-A* in the secondary cells, we stained for ABD-A protein in *iab-6*^*cocuD1*^ homozygous accessory glands. Fig 7 shows that in *iab-6*^*cocuD1*^ homozygous accessory glands, both ABD-A and ABD-B are undetectable. This is consistent with our RNA-seq results that show that the small amounts of *abd-A* transcript seen in wild type AGs drops ~4.5-fold in *iab-6*^*cocuD1*^mutants [36]. Thus, ABD-A does not seem to be regulated by the *msa* transcript in the AG and therefore, misexpression of ABD-A is not responsible for the *iab-6*^*cocu*^ phenotype.

Other known targets of *miR-iab-8* are the transcripts encoding the hox protein, ULTRABITHORAX (UBX) and its general cofactor EXTRADENTICLE (EXD) [29]. Both of these proteins have been shown to be misregulated in the CNS in *miR-iab-8* mutants and have been shown to be involved in the sterility phenotype associated with this deletion [29]. As our RNA-seq data suggest that both *Ubx* and *Exd* are transcribed in the accessory gland [36], we decided to check for the expression of both of these proteins in the secondary cells of wild type and *iab-6*^*cocuD1*^ mutants.

**Fig 8:**
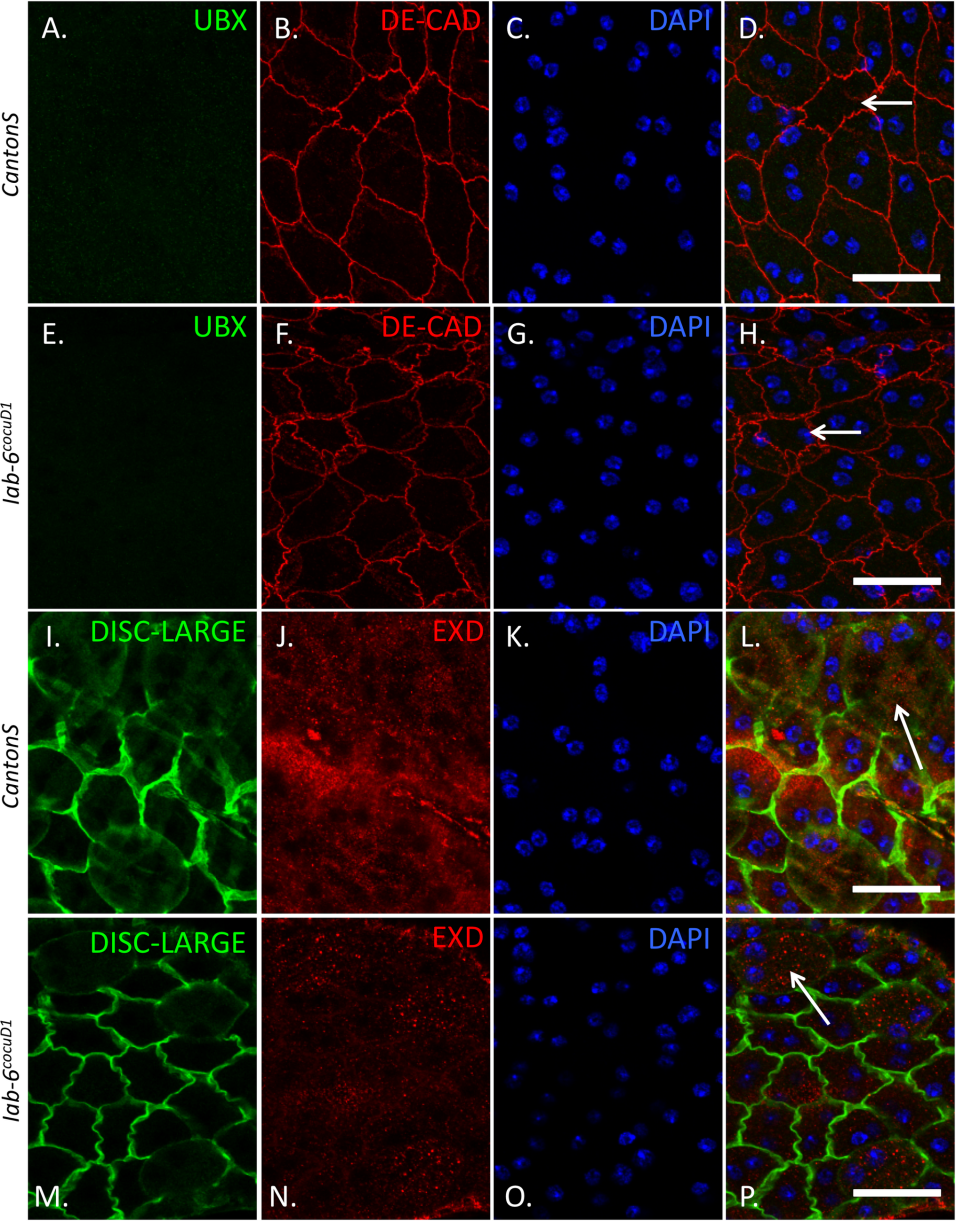
UBX and EXD level do not change by detectable levels in iab-6^cocuD1^ mutant accessory glands. UBX and EXD staining in *wild-type* or mutant secondary cells. Genotypes and detected proteins are labeled on the image. Accessory glands from CantonS (*wild-type*) (**A.-D.** and **I.-L.)**, or *iab-6*^*cocuD1*^*/ iab-6*^*cocuD1*^ (**E.-H.** and **M.-P.)**. Panels **A.-H.** are maximum intensity projections of glands stained for UBX (in green (**A.,D.** (merge of **A.-C.**),**E.** and **H.** (merge of **E.-G.**))), DE-Cadherin, an apical junction marker to show cell outlines (in red (**B., D.** (merge of **A.-C.**), **F.** and **H.** (merge of **E.-G.**))) and DAPI (in blue (**C., D.** (merge of **A.-C.**), **G.** and **H.** (merge of **E.-G.**))). Panels I.-P. are single slices from a Z-stack of glands stained for EXD (in red (**J.,L.** (merge of **I.-K.**), **N.** and **P.** (merge of **M.-O.**))), Disc-Large, a basal lateral cell junction marker to show cell outlines (in green (**I., L.** (merge of **I.-K.**),), **M.** and **P.** (merge of **M.-O.**))) and DAPI (in blue (**K., L.** (merge of **I.-K.**),), **O.** and **P.** (merge of **M.-O.**))). Arrows point to representative secondary cells (identified based on their characteristic pattern of DE-Cadherin staining) in each merged image. Scale = 50μm.

Immunostaining for either UBX and EXD failed to detect nuclear localized UBX or EXD protein in the secondary cells of both genotypes. While UBX protein could not be detected at all, EXD staining revealed a punctate cytoplasmic staining (Fig 8). Although we cannot rule out that the EXD staining is simply background, the conditions used are able to visualize EXD protein in control tissues (S3 Fig). We did not further investigate EXD as a potential target as the function of EXD is known to be nuclear and its nuclear localization is known to depend on its partner HTH [51] [52] [53] [54] [55]. Since our RNA-seq results indicate that HTH is not expressed in the secondary cells of wild type or *iab-6*^*cocuD1*^ mutants [36], any EXD misregulation is unlikely to cause the *iab-6*^*cocuD1*^ mutant phenotype.

Overall, from our ABD-A, UBX and EXD staining results, it seems that the known targets of the *iab-8* miRNA are not the cause of secondary cell defects seen in *iab-6*^*cocuD1*^ mutants. Given that the overexpression of these genes has been directly linked to the CNS mediated sterility phenotypes, we conclude that the primary targets of the *iab-8* miRNA are probably different in the CNS and the accessory glands.

## Discussion

Previously, we showed that the *iab-6*^*cocuD1*^ mutation removes a secondary cell specific enhancer for *Abd-B* and that this mutation causes the loss of *Abd-B* expression in the secondary cells. Furthermore, this mutation causes both a cytological phenotype in these cells and a reduction in the long-term post-mating response (LTR) in the mates of these mutant males [35]. Sequence analysis of a lncRNA discovered by large-scale RNA-seq [30], called *male-specific abdominal (msa)*, indicate that the ~1.1 kb *iab-6*^*cocuD1*^ deletion also removes the promoter and first exon of the *msa* lncRNA. By genetic and molecular analyses, we show that the expression of the *msa* lncRNA allows the *mir-iab-8* miRNA to be produced in the secondary cells and that this miRNA is responsible for some of the male’s ability to induce an LTR in his mate. Based on the data presented here, and from our previous work, it is clear that both *Abd-B* and the miRNA play overlapping, but not completely-redundant roles in secondary cell development; reduction of either *Abd-B* or the *mir-iab-8* miRNA alone show weaker phenotypes than the removal of both elements together (Fig 4, also see [35]).

That there is some non-redundancy between the effects of the ABD-B transcription factor and the *mir-iab-8* miRNA in secondary cells can also be seen with the LTR-mediating proteins CG1652 and CG1656. In both *iab-6*^*cocuD1*^ and *iab-6*^*cocuD1*^*/miR iab-8* mutants, we observe shifts in the gel-migration of these proteins relative to wildtype. However, both proteins migrate slightly differently in extracts of *iab-6*^*cocuD1*^*/miR iab-8* vs. extracts of *iab-6*^*cocuD1*^ homozygotes, indicating differences between these genotypes. We previously linked the subtle shifts in the migration pattern of CG1656 in *iab-6*^*cocu*^ mutants to changes in N-linked glycosylation [35]. PGNase F treatment of extracts from *iab-6*^*cocuD1*^ and *iab-6*^*cocuD1*^*/miR iab-8* mutants does not remove the subtle difference in SDS-PAGE migration, thus further highlighting the differences between the two genotypes and likely, the role of *Abd-B* vs the miRNA.

Earlier work from our lab and others have shown that *miR-iab-8* is important for male and female fertility through its role in the developing CNS [24] [27, 29]. In the early posterior CNS, *miR-iab-8* is important for the repression of specific homeotic genes and homeotic gene cofactors (*abd-A*, *Ubx*, *exd* and *hth*). Failure to repress these targets in the posterior CNS leads to male and female sterility, as males lack the ability to curl their abdomens for mating and females lack the ability to lay eggs [24]. These phenotypes seem to be due to CNS defects that prevent proper innervation of particular abdominal muscles [24] [29]. The results we present here suggest that *miR-iab-8* has at least some different primary targets in the secondary cells that are also needed for fertility. We have not yet been able to determine a primary targets for *miR-iab-8* in the secondary cells, though we have examined a number of genes that are upregulated in *iab-6*^*cocu*^ mutants and contain predicted miRNA binding sites [36]. Given that many miRNA loss-of-function phenotypes are thought to be caused by mildly affecting the expression level of many target genes [8, 56], our lack of success in finding a primary target could indicate that removing *miR-iab8* causes defects in the secondary cells through the mild overexpression of a network of targets.

As mentioned above, we now know that the *iab-8* miRNA plays a dual role in male fertility. One role is in the CNS and is accomplished through the regulation of the hox genes and their cofactors. Here, we have described a second role for *mir-iab-8;* in the secondary cells of the male AG, it plays an important fertility function that seems to be through the regulation of a different set of genes. Given the high conservation of *mir-iab-8* in arthropod hox complexes [57] and the conservation of its target sites in the homeotic genes and their effectors [58], we believe the homeotic function of the miRNA is likely its ancestral role. In this context, it is intriguing to speculate on the genesis of the *msa* transcript. In both flies and humans, male gonadal tissues have the highest levels of ncRNA expression [59] [60]. This has been suggested to reflect the high concentration of transcription factors in this tissue regulating cryptic promoters in intergenic regions [61] [62]. If the ncRNA provided advantages in fertility [59] [62–64] [65] it could be selected; perhaps such a scenario led to selection for secondary cell expression of *msa*.

The *msa* promoter seems to be tied to an *Abd-B* enhancer. Interestingly, *Abd-B* class hox genes have evolutionarily conserved functions in the male reproductive tissues. For example, *egl-5*, the *C*. *elegans Abd-B* ortholog is expressed in the male worm seminal fluid producing organs and is sufficient to induce markers associated with the male-specific, seminal fluid-producing cell fates in hermaphrodites [66]. In mammals, *Abd-B* class hox genes have been shown to be expressed in the male seminal fluid creating organs like the prostate and the seminal vesicle and have been shown to be critical for the production of secreted gene products. [67, 68]. Based on these conservations, it may be that the accessory gland function of *msa/miR-iab-8* arose from the co-opting of the *Abd-B* secondary cell enhancer (*iab-6*^*cocuD1*^) by a neighboring, potentially cryptic, promoter. As the creation of the *msa* transcript would not disturb hox gene regulation in the secondary cell (since its normal targets do not seem to be expressed in these cells), its appearance could have been tolerated, adding increased genetic flexibility for selection. One can imagine that transcripts of secondary cell-expressed genes whose repression was beneficial to male fertility might then have acquired/retained regulation *by miR-iab8*. Based on this, it would be particularly interesting to look for species that do not express the *msa* lncRNA and to examine predicted accessory gland targets for changes in *mir-iab8* binding sites.

## Materials and methods

### Fly stocks and media

Flies were raised at 25°C on standard yeast-cornmeal-agar or yeast-glucose-agar media and crossed using standard fly methods. Mutants and transgenic constructs used for this study include: *Δmir-iab-8*^*121−8*^[24], *iab-6*^*cocuD1*^, *iab-6*^*cocuD5*^, *Abd-B-Gal4*, *UAS-nGFP/CyO; iab-6*^*cocuD1*^ (this study), *Df(P9)* (Lewis, 1978), *iab-4*^*186*^, *iab-6*^*11*^ [39], *Abd-B*^*D16*^[69]*, miR-iab-8 sensor [29], miR-iab-8 sensor; iab-6*^*cocuD1*^, *Fab-8*^*64*^ [41], *iab-3*^*86A*^[40], *iab-5*,*6*^*rescue*^ [70].

### Molecular biology and generation mutations sued in this study

All molecular biology was performed using enzymes from NEB (Ipswich, MA USA) or Promega (Promega). *The iab-6*^*cocuD1*^ mutation was created in a fashion similar to the original *iab-6*^*cocu*^ mutation in Gligorov *et al*. [35, 36] [35]. In short, a kanamycin cassette was amplified using the following primers: FI (5’-GGCAGCACGAATAGTTTAGTTTATTTTAGCCATAGCTCAAGAACGACAGCGAATACAAGCTTGGGCTGCAGG-3’) and RI (5’-GGTGAATAATTTTTATTGCCGTAAATCACTGTGTCAATTGTGGTTGTAATCTCGCCCGGGGATCCTCTAGAG-3’). This cassette was then used to recombineer a plasmid that could be used for the InSIRT technique to target the *iab-6* domain for site specific mutation [70].

The mCherry reporter in exon 3 of the *iab-8 lncRNA* was made using by site specific integration into an attP site within the BX-C that removes the *iab-6* domain (*iab-5*,*6*^*C*.*I*^*)* [70]. The integration construct was made by two successive recombineering steps using the Counter-Selection BAC Modification Kit (Gene Bridges) on pKsY-iab6H [70]. Amplification of the rpsl-neo cassette with homology regions flanking exon 3 was performed using the primers: *Rpsl*-neo F: TATACTTTATGCCCTTCCAGTTTGATTACACATCGACCCCTGGAGCGAGCCAAACGGCCTGGTGATGATGGCGGG and *Rpsl*-neo R:TATGAAATATGTTAAGATGGAGACTCAC-CTGATGCAGCTGCCGTCGGGTTAAGTCTCAGAAGAACTCGTCAAGAA. The resulting PCR fragment was used to insert an rpsl-neo cassette into exon 3. Next, mCherry was amplified using the primers: MCherry F: TATACTTTATGCCCTTCCAGTTT-GATTACACATCGACCCCTGGAGCGAGCCAAACGGTACCATGGTGAGCAAGGG and MCherry R: TATGAAATATGTTAAGATGGAGACTCACCTGATGCAGCTGCCGTCGGG-TTAAGTCGCCCCAAGGGGTTATGCTAG. The resulting PCR fragment was used to recombineer the rpsl-neo exon 3 construct to replace the rpsl-neo with the exon-3-mCherry fusion. This plasmid was then integrated into the bithorax complex using the InSiRT technique [70].

*msa* deletions and inversions were created using the CRISPR-Cas9 system. Guide RNAs were created in the embryo from plasmids containing guide sequences and by injecting U6-promoter target guide-gRNA fusion plasmids. The templates for the guide RNA plasmids were created as g-blocks (IDT, Coralville, Iowa USA) and cloned into pGemTeasy (Promega). The target guide sequences are GGTGGCAAAATATCAAACAA(TGG) and GATGAGAGCAATAGTAGAAG(AGG)(PAM sequences in parentheses). Guide plasmids were injected using standard microinjection procedures into *vasa-Cas9*, *lig4*^*169*^ flies. The vasa-Cas9 transgene was made by PCR. The *vasa* promoter and 5’ UTR was made by PCR using primers 5’vasapromS: 5’ GATATCTTTGGACACGTGGCATAAACAAGCC 3’ and NcoI vasa5’UTRAS: 5’CCATGGTATTGATATTTTTTTTTTAATTTGGCCTGCCTTTC3’. The vasa 3’UTR was made by PCR using the primers NcoI-ApaI vasa3’UTRS: 5’CCATGGAAGGGCCCAATGTATGGACATAGATTTCAAATAATTAAATGTAATGC3’ and SacI vasa3’UTRAS: 5’GAGCTCAACACGAAGAGCAGCAGTGTGGTGG3’. First the vas promoter/5’UTR was cloned into pGemTeasy. Clones in the proper direction were then cut by ApaI, blunt ended using Mung bean exonuclease (New England Biolabs), then cut with EcoRV and religated. The Vasa3’UTR PCR fragment was then cut with NcoI and SacI. The pGemTeasy vector with the vasa promoter/5’ UTR was cut with NcoI and SacI and the cut vasa 3’UTR fragment was cloned into it. The Vasa 5’3’UTR construct was then placed into pTnT (Promega). The vasa 5’3’ construct was then cut with NotI, blunt ended and religated. The Cas9 coding sequence was excised from plasmid X260 (a kind gift of Feng Zhang)[71] using NcoI and NotI and ligated into the pTnT vasa construct cut with NcoI and PspOMI. The whole vasa-Cas9 cassette was then removed by a partial EcoRI digest and cutting with XhoI. This fragment was then ligated into a pATTB cut with EcoRI and XhoI. The resulting vector was then injected into the ZH2A attP platform (FlyC31.org[72]). After isolating transformants, the lines were recombined with a *lig4*^*169*^ mutation [73].

### Immunofluorescence and microscopy

For imaging accessory glands, 3–5 day old virgin male flies were dissected in Grace’s Insect Media and fixed for 20 minutes using 3.7% formaldehyde in PBST or Grace’s Insect Media. Samples were blocked in PBST buffer containing 5% horse serum. Primary antibodies were incubated in the blocking solution overnight at 4°. Secondary antibodies were incubated for 1–2 hours at room temperature in blocking solution. All washes were done with PBST and generally consist of 3 rinses of 30 seconds followed by 3 washes of 20 minutes. All samples were mounted in Vectashield with DAPI (Vector Labs). Mouse anti-Abd-B (Developmental Systems Hybridoma Bank) supernatant was pre-absorbed against Drosophila embryos and then diluted to 1:100 before use. Goat anti-ABD-A (Santa Cruz Biotechnology) and Goat anti-Exd (Santa Cruz Biotechnology) were both used at a 1:50 dilutions. Mouse Anti-Ubx (Developmental Systems Hybridoma Bank) was used at a 1:10 dilution. Secondary antibodies were Alexa Fluor 488 or 555 antibodies (Invitrogen AG) and were used at a 1:1000 dilution. GFP fluorescence was visualized directly.

Microscopy was performed on a Zeiss Axioplan fluorescence microscope using an X-Lite 120 lamp or a Zeiss LSM 700 confocal microscope.

### Western blots

Accessory glands were dissected from 3–5 day old virgin males, placed into tubes with SDS-sample buffer, homogenized, and boiled for 5 min. Extract representing the equivalent of one male’s accessory glands was loaded onto 12% or 4–20% SDS-PAGE gels, purchased from (Invitrogen/Thermo Fisher Scientific). The proteins were run slowly (20–30 volts) through the stacking gel and first half of the separating gel. Later, when the proteins were in the separating gel, the voltage was turned up to at 100V. Transfer of the proteins onto PVDF membranes was performed using a Biorad wet transfer cell for 1 hour at 100V. Antibodies used for the western blots were affinity purified rabbit anti-CG1656 (1:500 dilution) [74], affinity purified rabbit anti-CG1652 (1:250) [74] and rabbit anti-Acp36DE (1:30000)[75]. Secondary antibodies were goat anti-rabbit antibodies conjugated to either alkaline phosphatase (Biorad) or horseradish peroxidase (Promega). Signals for the alkaline phosphatase conjugated antibodies were visualized according to the NBT/BCIP staining kit (Roche), while peroxidase conjugated antibodies were visualized according to the Supersignal West Pico PLUS Chemiluminescent Substrate kit (Invitrogen/Thermo Fisher Scientific). PGNase treatments of AG protein extracts were performed as in Gligorov et al. {Gligorov, 2013 #176}

### Quantification of vacuole size

Three to four day old virgin males, raised at 25°C of the following genotypes were collected: *Abd-B-Gal4*, *UAS-nGFP/CyO; iab-6*^*cocuD1*^[36], *Abd-B-Gal4*, *UAS-nGFP/+; iab-6*^*cocuD1*^/ *+*, *Abd-B-Gal4*, *UAS-nGFP/+; iab-6*^*cocuD1*^/*Δmir-iab-8*^*121−8*^ and *Abd-B-Gal4*, *UAS-nGFP/+; iab-6*^*cocuD1*^/*Abd-B*^*D16*^. The accessory glands were dissected in Grace’s Insect Media and fixed in 3.7% formaldehyde for 20 minutes. After washing with PBST, the accessory glands were mounted on slides in vectashield mounting medium. Vacuoles were visualized using GFP fluorescence, captured by a Leica LSM700 confocal microscope.

Vacuoles were measured using the ImageJ software; the stacks were examined and the area of every vacuole seen in a cell was measured in the stack at its point of largest diameter. For each genotype, the vacuoles of whole cells were measured from a number of different glands. The recorded data were then statistically analysed using Kruskal-Wallis analysis of variance with *post-hoc* Dunn’s tests using the Prism 7.0 software (Graphpad Software, Inc.).

### Fertility/fecundity assays (FFA)

Fertility/fecundity assays were performed as previously described [35] with Wilcoxon non-parametric tests used to compare results for mates of different genotypes in total and on individual days. rmANOVA was used to evaluate overall 10-day trends. All statistical analysis was performed with the JMP9 software [76]. For each genotype, mates of between 15 and 22 males were tested (see figure legend for exact numbers).

### Receptivity assays

Receptivity assays were performed as described in [35]. Comparisons of remating frequencies between females mated to different genotype males was evaluated using a Wilcoxon ranked sums test (WRST) using JMP9 software [76]. For each genotype, mates of between 15 and 22 males were tested (see figure legend for exact numbers).

## Supporting information

S1 Fig Abd-B expression in different fly lines used in this study. Shown are AGs from Canton S *(****A*., *B*.***)*, *iab-6*^*cocuD1*^*/ iab-6*^*cocuD1*^*(****C*.,*D*.***)* or *iab-6*^*cocuD1*^*/mir-iab-8 (****E*.**, ***F*.***)* males, stained for Abd-B (***A*.**, ***C*.** and ***E*.** in green) and DAPI (***B*.**, ***D*.** and ***F*.** in blue). Scale = 25μm.
(TIFF)

S2 Fig A lncRNA is plays a role in the development of the accessory gland DIC images. Accessory gland phenotypes associated with BX-C mutations using Nomarski microsocpy. Each panel shows the accessory gland from a male hemizygous for a BX-C chromosomal break (shown in Fig 1). In each panel, a single secondary cell is indicated by a dashed line. In wild type secondary cells ***A*.**, vacuoles and nuclei can be seen as large “fried egg”-like structures within the cell. In mutants defective in vacuole formation, like *iab-6*^*cocuD1*^ (***B*.**), the secondary cells take on a “grainy” appearance and smaller ball-like structures become visible. Genotypes are labeled in each panel: ***A*.** A wild-type accessory gland, ***B*.** *iab-6*^*cocuD1*^*/ iab-6*^*cocuD1*^, ***C*.** *iab-3*^*86A*^*/Df(3R)P9*, ***D*.** *iab-6*^*11*^*/Df(3R)P9*, ***E***. *Fab-8*^*64*^*/Df(3R)P9 and* ***F*.** *iab-4*^*186A*^*/Df(3R)P9*. Note the large egg-like structures in ***A*.**, ***C*.** and ***E*.**, and the smaller ball-like structures in ***B*.**, ***D*.** and ***F***. Scale bar = 25μm.
(TIFF)

S3 Fig UBX and EXD staining in control tissues. Panels are labeled in the figure. UBX can be seen in the abdominal lobe of the brain in the expected expression pattern ([29]). Cytoplasmic EXD can be seen in the central wing disc as reported in ([54]). Scale = 50μm.
(TIFF)
