# Supplementary figures and images for: The lncRNA male-specific abdominal plays a critical role in Drosophila accessory gland development and male fertility
Source: PLoS Genet. 2018 Jul 16;14(7):e1007519. doi: 10.1371/journal.pgen.1007519 (PMC6067764; doi:10.1371/journal.pgen.1007519)

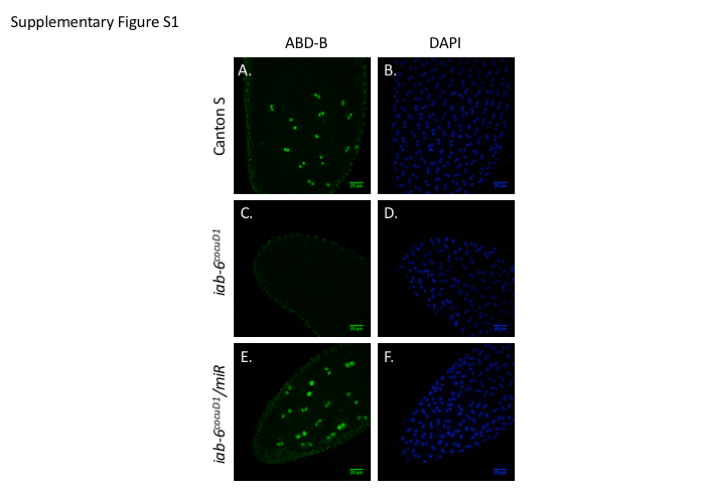

Supplement: S1 Fig — Shown are AGs from Canton S (A., B.), iab-6cocuD1/ iab-6cocuD1(C.,D.) or iab-6cocuD1/mir-iab-8 (E., F.) males, stained for Abd-B (A., C. and E. in green) and DAPI (B., D. and F. in blue). Scale = 25μm. (TIFF) [file pgen.1007519.s001.tiff]

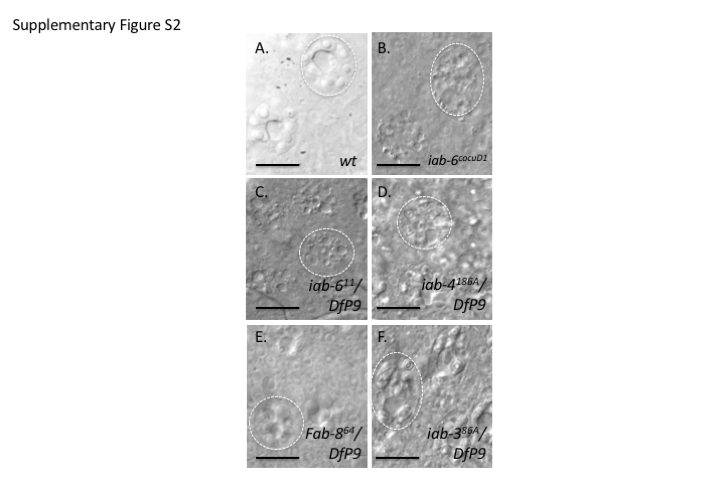

Supplement: S2 Fig — Accessory gland phenotypes associated with BX-C mutations using Nomarski microsocpy. Each panel shows the accessory gland from a male hemizygous for a BX-C chromosomal break (shown in Fig 1). In each panel, a single secondary cell is indicated by a dashed line. In wild type secondary cells A., vacuoles and nuclei can be seen as large “fried egg”-like structures within the cell. In mutants defective in vacuole formation, like iab-6cocuD1 (B.), the secondary cells take on a “grainy” appearance and smaller ball-like structures become visible. Genotypes are labeled in each panel: A. A wild-type accessory gland, B. iab-6cocuD1/ iab-6cocuD1, C. iab-386A/Df(3R)P9, D. iab-611/Df(3R)P9, E. Fab-864/Df(3R)P9 and F. iab-4186A/Df(3R)P9. Note the large egg-like structures in A., C. and E., and the smaller ball-like structures in B., D. and F. Scale bar = 25μm. (TIFF) [file pgen.1007519.s002.tiff]

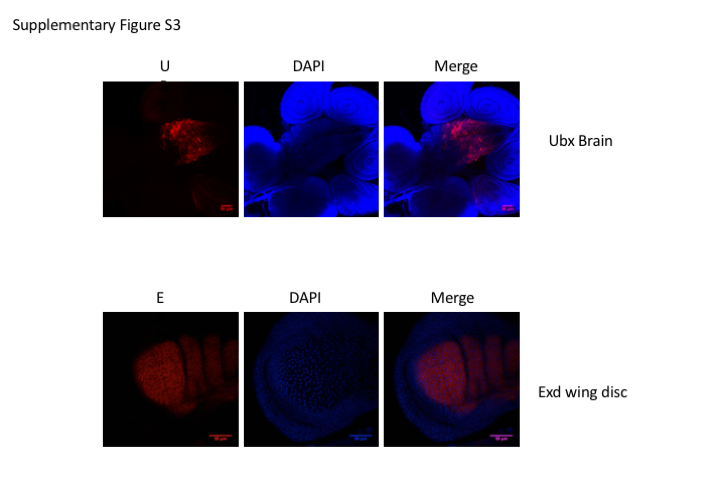

Supplement: S3 Fig — Panels are labeled in the figure. UBX can be seen in the abdominal lobe of the brain in the expected expression pattern ([29]). Cytoplasmic EXD can be seen in the central wing disc as reported in ([54]). Scale = 50μm. (TIFF) [file pgen.1007519.s003.tiff]
